# Joint Effects of Prenatal Folic Acid Supplement with Prenatal Multivitamin and Iron Supplement on Obesity in Preschoolers Born SGA: Sex Specific Difference

**DOI:** 10.3390/nu15020380

**Published:** 2023-01-12

**Authors:** Qing Lu, Esben Strodl, Yang Liang, Li-Hua Huang, Bing-Jie Hu, Wei-Qing Chen

**Affiliations:** 1 Department of Epidemiology, School of Public Health, Sun Yat-Sen University, Guangzhou 510080, China; 2 School of Psychology and Counselling, Queensland University of Technology, Brisbane, QLD 4059, Australia; 3 School of Basic Medical Science, Guangzhou Medical University, Guangzhou 511436, China; 4 School of Health, Xinhua College of Guangzhou, Guangzhou 510080, China

**Keywords:** obesity, prenatal supplement, folic acid, iron, multivitamin, preschoolers, SGA

## Abstract

Prenatal maternal nutrient supplementation has been reported to be associated with offspring obesity, but the reports are inconsistent and have mainly ignored the differences between the total children population and children born small for gestational age (SGA). This study aimed to examine the joint effects of folic acid, iron, and multivitamin supplementation during pregnancy on the risk of obesity in preschoolers born SGA. A total of 8918 children aged 3–6.5 years born SGA were recruited from Longhua District in Shenzhen of China in 2021. Their mothers completed a structured questionnaire about the child’s and parents’ socio-demographic characteristics, maternal prepregnant obesity, and mothers’ prenatal supplementation of folic acid, iron, and multivitamin. In addition, the children’s current weight and height were measured by trained nurses. Logistic regression models were used to analyze the associations between prenatal supplementations and the current presence of childhood obesity. After controlling for potential confounders, the results of the logistic regression analysis showed that prenatal supplement of folic acid (OR = 0.72, 95% CI = 0.55~0.93) was associated with a lower likelihood of being an obese preschooler born SGA. In contrast, the ingestion of multivitamin or iron supplements during pregnancy did not seem to be related to the likelihood of childhood obesity in preschoolers born SGA. Moreover, cross-over analysis of prenatal folic acid and multivitamin obtained significant negative associations of prenatal folic acid supplement only (OR = 0.73, 95% CI = 0.55~0.97) and combination supplement of folic acid and multivitamin (OR = 0.67, 95% CI = 0.50~0.90) with obesity of preschoolers born SGA; while the cross-over analysis of prenatal folic acid and iron observed significant negative associations between obesity of preschoolers born SGA and a combination supplement of folic acid and iron (OR = 0.70, 95% CI = 0.52~0.96). Furthermore, the aforementioned significant associations were only found in girls and not in boys when the analyses were stratified by sex. Our findings suggest that the prenatal folic acid supplementation may decrease the risk of obesity in preschool girls born SGA, and that this effect may be modified by prenatal multivitamin or iron supplementation.

## 1. Introduction

Obesity is one of the most severe public health concerns in the world, ranking fourth among risk factors for mortality and accounting for 4.72 million deaths worldwide in 2017 [1]. This is concerning given that the global prevalence of obesity in children and adolescents increased from 0.7% in 1975 to 5.6% in 2016 for girls and from 0.9% to 7.8% for boys [2]. For Chinese preschoolers, overweight and obesity prevalence has been estimated as high as 8.4% and 4.2%, respectively [3]. While obesity is mainly a risk factor for the development of chronic diseases in adulthood [4], childhood obesity is a major public health issue given that obese children are very likely to remain obese into adulthood [5]. Given this trajectory, reported lifetime healthcare cost and loss of productivity of an obese child or adolescent is high (approximately EUR 149,000) compared with a normal weight child or adolescent [6]. Early prevention and intervention of childhood obesity is, therefore, critical for both the individual and society. To guide the development of effective prevention programs it is important to identify significant risk and protective factors for childhood obesity.

It has been well documented that childhood obesity Is caused by synergistical effects between genetic, prenatal, and postnatal factors. A body of evidence has revealed that specific genes correlate with childhood obesity. For example, a meta-analysis reported that a fat-mass and obesity-associated gene (FTO) increased the risk of obesity among children [7]. Similarly, a genome-wide association study in China linked single nucleotide polymorphisms near SEC16B, RBJ, CDKAL1, TFAP2B, MAP2K5, and FTO to childhood obesity [8]. In addition, there are several genes related to energy metabolism or appetite regulation, such as melanocortin 4 receptor gene leptin and the leptin receptor gene, that have been associated with childhood obesity [9,10]. With regard to prenatal factors, studies have shown that deficient maternal exercise, exposure to environmental tobacco smoke, unhealthy dietary pattern, prepregnancy maternal BMI maternal weight gain during pregnancy, as well as poor birth outcomes, such as low birth weight (LBW) or small for gestational age (SGA) and preterm birth, are all associated with an increased likelihood of childhood obesity [11,12,13,14,15]. For postnatal factors, prior research indicates that childhood obesity is positively associated with early-life rapid weight gain of babies born SGA [16]. In addition, artificial feeding in early life—in the first six months—unbalanced nutrition, short sleep duration, and insufficient physical activity in childhood are also all related to childhood obesity [11,12,13].

SGA, defined as a birth weight less than the 10th percentile for that gestational age, is typically considered a fetal growth restriction (FGR) at birth [17]. Epidemiological studies have shown that SGA is associated with neurodevelopmental delay [18], obesity [19,20], and other cardiometabolic risk factors in childhood [21], and with chronic diseases in adulthood, such as cardiovascular diseases and type 2 diabetes [22]. Based on the Developmental Origins of Health and Disease Hypothesis (DOHaD) [19], an undesirable intrauterine environment increases the incidence of SGA and later high-risk metabolic patterns through possible mechanisms of placental dysfunction [17] and epigenetic modification (e.g., DNA methylation) [23,24].

Increasing evidence indicates that prenatal nutrients play a crucial role in fetal growth, offspring’s development, and the likelihood of experiencing disease [11,25,26,27]. For example, several experimental studies have found that prenatal micronutrient supplements improved fetal growth [17,28,29,30] and reduced the incidence of SGA in fetuses diagnosed as FGR [31]. A randomized trial in Nepal [27] suggested that a prenatal micronutrients supplement might promote the metabolic status in young children. However, another study by Sauder et al. found that prenatal multivitamin use might slow the growth of an offspring during infancy [32]. Recently, a prospective study showed an inverse association between prenatal maternal iron supplementation and the infant’s fat mass at birth, and 3 months and 6 months post-birth [33].

Folic acid is widely used to prevent neural tube defects [34]. Additionally, two population-based studies in China reported that maternal folic acid supplementation during pregnancy reduced SGA and low birth weight [35,36]. An animal trial found that prenatal folic acid or methyl donor supplements could prevent the offspring from developing obesity [37]. A study of 4449 school-age children reported that a relative higher folate concentration in maternal plasma during pregnancy decreased the risk of the offspring being overweight [38]. Conversely, a study by Yajnik et al. suggested higher circulating concentrations of maternal erythrocyte folate during pregnancy increased offspring adiposity [39].

It has been recognized that children born SGA have a higher tendency to develop obesity; however, not all individuals born SGA develop obesity. Moreover, prenatal supplements of folic acid, multivitamin, and iron may be associated with the likelihood of offspring obesity, but the results are inconsistent. Furthermore, most studies have mainly focused on the total population of children, and not just on children with SGA. As such, an unanswered question remains as to whether prenatal supplementation of folic acid, multivitamin, and iron affects the development of obesity in offspring born SGA. Therefore, this study aimed to investigate the joint effects of prenatal folic acid supplementation with prenatal multivitamin and iron supplementation on obesity in Chinese preschoolers born SGA.

## 2. Materials and Methods

### 2.1. Study Population

Participants of this research were recruited from all of the kindergartens in the Longhua District of Shenzhen, China, with a total of 69,639 child–mother dyads recruited. The study was approved by the Ethics Committee of School of Public Health, Sun Yat-sen University in Guangzhou, China. Written informed consent was obtained from the mothers of all of the preschoolers. The study was carried out in accordance with the Declaration of Helsinki.

This paper only included 8919 preschoolers born singleton and classified as SGA based upon being under the 10th percentile of Chinese birth weight for gestational age [40]. Due to missing data, 143 child–mother pairs were excluded for lacking information of weight or height, 214 pairs were excluded for missing paternal age at childbirth, 445 for missing maternal age at childbirth, 12 for missing paternal education level, and 88 for missing maternal prepregnant BMI. Finally, a total of 8016 child–mother pairs were included in the final analysis.

### 2.2. Data Collection

The enrolled mothers were asked to complete a self-report structured questionnaire collecting the socio-demographic characteristics of the child and the parents (such as age, gender, maternal prepregnant BMI, parents’ marital status, parents’ education level, family income, and single child or not), prenatal maternal supplementations of multivitamin, folic acid, and iron, and birth-related variables (mode of delivery, gestational age, and birth weight).

### 2.3. Measurement of Prenatal Maternal Nutrient Supplementations

Based upon previous research [41,42,43,44], we collected the data on folic acid, multivitamin, and iron supplementations during pregnancy using the following questions: (1) Did the mother take folic acid during pregnancy? (2) Did the mother take a multivitamin during pregnancy? (3) Did the mother take iron during pregnancy? The answers for each question were: 1 for “yes”, and 0 for “no”.

### 2.4. Measurement and Definition of Obesity

Standardized measurements of the weight and height of the child were taken by trained nurses from Longhua Maternity & Child Healthcare Hospital. A portable electronic weight scale (fractional value = 0.01 kg) was used to measure the child’s weight by placing the scale on level ground and asking the preschoolers to stand in the center of the scale bareheaded, barefooted, and wearing close-fitting light clothes. After the value stabilized, the nurses read and recorded the measurement accurate to 0.1 kg. Height was measured by column human altimeter (fractional value = 0.1 cm). The column human altimeter was placed vertically against the wall on horizontal ground. Preschoolers were asked to stand on the pedal, with heels close and feet separated by a 60-degree angle, chest lifted, abdomen pulled in, and eyes straight ahead. Nurses slid the slider to the apex of the measured child’s skull, and read the measurements with their line of sight the same height as the slide board.

Body mass index (BMI) was calculated by dividing weight in kilograms by height in meters squared. We employed BMI reference values based upon the normative data obtained from two national representative cross-sectional surveys in China: The National Growth Survey of Children under 7 years in the Nine Cities of China in 2005 and The Physical Fitness and Health Surveillance of Chinese School Students in 2005. The researchers used the LMS method to smooth the curve of BMI, calculated values of percentile, and generated the screening cut-offs of obesity in Chinese children by integrating with adult cut-offs for obesity at 18 years in China [45]. Based on the aforementioned investigation in China, this research defined obesity as a BMI equal to or greater than the reference values for sex and age [45].

### 2.5. Potential Confounding Variables

In the light of previously findings [46,47], potential confounding variables included in this study were the child’s sex, child’s age, prepregnant maternal BMI, mode of delivery, parents’ marital status, parents’ age at the childbirth, parents’ education level, single child or not, and family income.

### 2.6. Statistical Analyses

Means and standard deviations (SD) were used to describe the continuous variables, while frequencies and percentages were used to describe the categorical variables. Categorical covariates and numeric covariates were compared using a chi-square test and *t*-test, respectively.

A series of binary logistic regression analyses were conducted to examine the association of maternal supplementations of multivitamin, folic acid, and iron during pregnancy with obesity in SGA children after adjusting for the potential confounding variables. Their multiplicative interaction effects on obesity were tested by establishing multiplicative terms in logistic regression models with the interaction of odds ratio (IOR) used to indicate the strength of multiplicative interaction effects. Moreover, crossover analyses were employed to assess the additive interaction effects and modification effects among the combination of the three nutrients. Adjusted odds ratios (AOR), the relative excess risk due to interaction (RERI), and the attributable proportion due to interaction (AP) were used to indicate the strength of these effects.

Furthermore, after stratification by sex, the aforementioned analyses were repeated to evaluate sex-specific associations of prenatal supplementations with obesity in SGA preschoolers.

All statistical analyses were performed in RStudio versions 4.1.2 (Poist, BOSTON, MA, USA) and two-tailed *p*-values < 0.05 were deemed statistically significant.

## 3. Results

### 3.1. Characteristics of Participants

Table 1 presents the demographic characteristics and pregnancy condition of the participants. The mean age was 4.85 years (SD = 0.84) old for the children, 30.55 years (SD = 4.83) old for their fathers and 28.44 years (SD = 4.27) old for their mothers, respectively. The mean birthweight was 2.72 kg (SD = 0.41) and the mean gestational age at childbirth was 39.65 weeks (SD = 1.90). There were only 204 mothers (2.54%) with acknowledged gestational hypertension history. Over 60% of the preschoolers were male. The mean value was 15.33 kg/m^2^ (SD = 2.18) for children’s BMI, 18.07 kg (SD = 3.64) for children’s weight, 108.34 cm (SD = 7.69) for children’s height, and 20.31 kg/m^2^ (SD = 2.81) for their mothers before pregnancy, respectively. There were only 119 children (1.48%) born to obese mothers. Most mothers (99.09%) and fathers (98.52%) were married. The proportion of mothers and fathers with high school education and above was 83.16% and 85.32%, respectively. More than half of participants had a sibling(s) (63.91%) and a high household income (82.92%).

The prevalence of obesity among the preschoolers was 8.76%. Significant differences of the following characteristics were observed between obese and normal weight preschoolers, including the child’s sex, maternal prepregnant obesity/BMI, paternal education level, monthly household income, and being a single child.

### 3.2. Associations between Prenatal Supplementation of the Micronutrients and Obesity in Preschoolers Born SGA

After controlling for the potential confounding variables, the results of the logistic regression analysis showed that prenatal folic acid supplement was significantly and negatively associated with obesity in preschoolers born SGA (AOR = 0.72, 95% CI = 0.55~0.93). No significant associations were found between prenatal maternal iron and multivitamin supplementation and childhood obesity (Table 2).

### 3.3. Combination Effects of Maternal Micronutrients Supplementation during Pregnancy on Obesity in Preschoolers Born SGA

Table 3 presents the combination effects of maternal micronutrients supplementation during pregnancy on obesity in preschoolers born SGA. The results of the crossover analyses indicated that a maternal supplement of a combination of folic acid and multivitamin (AOR = 0.67, 95% CI = 0.50~0.90), as well as maternal supplement of a combination of folic acid and iron (AOR = 0.70, 95% CI = 0.52~0.96) significantly decreased the risk of obesity in preschoolers born SGA; while only maternal folic acid supplementation during pregnancy decreased the risk of obesity with significance (AOR = 0.73, 95% CI = 0.55~0.97) in the crossover analysis on a combination of folic acid and multivitamin, and marginal significance (AOR = 0.77, 95% CI = 0.57~1.04) in the crossover analysis on a combination of folic acid and iron, respectively. There was no significant multiplicative and additive interaction between prenatal maternal supplementation of the three nutrients on obesity in preschool children born SGA.

### 3.4. Combination Effects of Maternal Micronutrients Supplementation during Pregnancy on Obesity in Preschoolers Born SGA

Table 4 presents the results of the stratification analysis by sex. After controlling for the potential confounding variables, the results of the logistic analysis showed that prenatal maternal folic acid supplement was significantly and negatively associated with obesity in preschoolers born SGA (AOR = 0.57, 95% CI = 0.36~0.90) in girls, but not in boys.

Table 5 presents the combination effects of maternal micronutrients supplementation during pregnancy on obesity in preschoolers born SGA stratified by sex. The results of the crossover analyses indicate that combining the supplements of folic acid and multivitamin (AOR = 0.55, 95% CI = 0.33~0.92), as well as combining the supplements of folic acid and iron (AOR = 0.51, 95% CI = 0.30~0.86) significantly decreased the risk of obesity in girls, while only maternal folic acid supplementation during pregnancy decreased the risk of obesity with significance in the crossover analysis on a combination of folic acid and multivitamin, as well as in the crossover analysis on a combination of folic acid and iron in girls, and their ORs were 0.50 (95% CI = 0.31~0.82) and 0.57 (95% CI = 0.35~0.94), respectively. There was no significant multiplicative and additive interaction between prenatal maternal supplement of three nutrients on obesity in both male and female preschool children born SGA.

## 4. Discussion

To the best of our knowledge, this is the first study to examine the joint effects of prenatal maternal supplements of multivitamin, folic acid, and iron on childhood obesity in a large sample of Chinese preschoolers born SGA. Our results show that maternal folic acid supplementation in pregnancy is significantly and negatively associated with obesity in preschool girls born SGA, but not in preschool boys born SGA. Moreover, this association may be modified by a prenatal supplement of iron or multivitamin.

### 4.1. Associations of Prenatal Maternal Supplementation of Folic Acid with Preschool Obesity in SGA

There have been several previous studies examining the associations between maternal folate concentration and children obesity, but they have failed to yield consistent findings [48]. For example, a study involving 4449 school-age children reported that an increase in one standard deviation score (SDS) on maternal serum concentrations of folate was associated with a decreased BMI in the offspring (−0.04 SDS, 95% CI = −0.08~−0.01) [38]. Similarly, a cohort study with 1517 mother–child dyads in Boston, USA further showed that pregnant mothers with the lowest quantile of plasma folate concentration (6.64~20.36 nmol/L) were significantly more likely to have offspring who were overweight or obese (OR = 1.45, 95% CI = 1.13~1.87) [49]. In contrast, a study in Pune, India found that maternal erythrocyte folate concentrations at 28 weeks of pregnancy was positively related to the offspring’s fat mass at 6 years old [39]. Interestingly, our study found that a prenatal maternal folic acid supplement reduced the risk of obesity in preschool girls born SGA. Inconsistent findings of these studies might arise from differences of prenatal maternal folic acid measurement, and the selection of adjusted confounders in these studies.

Our findings raise the question of what the biomedical mechanism may be by which prenatal supplements of folic acid affect childhood obesity? Folic acid is pivotal for cellular growth, nucleic acid synthesis, and as a classic one-carbon metabolite [50]. It is also important in the synthesis of S-adenosylmethionine, which is the main methyl donor and affects DNA methylation reactions, gene expression, and chronic disease development [51,52,53,54]. Prior studies have demonstrated the influence of prenatal maternal folic acid intake on offspring’s DNA methylation of IGF2, leptin, and retinoid X receptor-α gene, which have all been shown to be related to growth, energy metabolism, and appetite regulation that affected energy balance and obesity [52,55]. For example, hypomethylation of leptin is recognized as decreasing the risk of obesity through increasing leptin expression and restraining appetite [52]. However, a rat model has revealed that maternal intake of folic acid, at more than the recommended dose, may be associated with a lower expression of leptin receptor and proopiomelanocortin, failing to suppress food intake [56]. Moreover, a prenatal supplement of folic acid appears to maintain normal placenta structure and functions through anti-inflammatory effects [57,58], and ultimately works in programming chronic diseases [59]. Unfortunately, we are far from confirming the precise biomedical mechanisms that explain the associations identified in our study, so further studies are needed to answer questions about causal mechanisms as well as dose-dependent responses.

### 4.2. Modification Effects of Prenatal Supplement of Iron or Multivitamin on Associations between Prenatal Maternal Supplementation of Folic Acid and Obesity

A systematic analysis indicated that multi-micronutrient (MMN) containing iron and folic acid could reduce more preterm birth, SGA, and low birth weight (LBW) more than supplementation of iron or folic acid alone did in pregnant mothers [42]. This finding has been supported by an intervention study by Yijun Kang et al., who also reported similar results [60]. Additionally, a double-blind randomized controlled trial in China found that the incidence of preterm birth was 4.2% for prenatal supplementation of iron and folic acid and 4.6% prenatal supplementation of folic acid, respectively [61]. In line with these previous findings, our study found that maternal supplementation of both folic acid and iron in pregnancy reduced the risk of obesity among preschoolers born SGA in girls with statistical significance, and in boys without significance, compared with maternal supplementation of folic acid during pregnancy only. The mechanisms for prenatal supplement of iron strengthening the effect on prenatal supplementation of folic acid reducing the risk of childhood obesity may be related to iron supplementation affecting the transcription of folic acid transporters [62] and regulating the metabolism of folate-activated one-carbon units [63].

There is no report on the combination effects of prenatal supplement of folic acid and multivitamin on childhood obesity. Our study found that maternal supplementation of both folic acid and iron in pregnancy significantly reduced the risk of obesity among preschoolers born SGA in girls, but its extent was less than maternal supplementation of folic acid only. Regarding the mechanisms for prenatal multivitamin supplement modifying the prenatal supplementation of folic acid reducing the risk of childhood obesity, vitamin B_2_ could promote the metabolism of folate in body [64], while vitamin B_6_ and vitamin B_12_ could modulate one-carbon metabolism to affect DNA methylation [65,66]. All of these evidences implicated that B-vitamins contained in a multivitamin might modify prenatal folic acid supplement, decreasing the risk of offspring’s obesity.

Of course, further in-depth animal experiments and epidemiological research are needed to clarify the mechanisms for prenatal supplement of iron or multivitamin modifying prenatal maternal supplementation of folic acid reducing the risk of obesity in offspring.

### 4.3. Sex Specific Differences of Associations between Prenatal Supplement of Folic Acid, Iron, and Multivitamin and Offspring’s Obesity

There have been several studies investigating the sex-specific effects of prenatal folic acid supplement on offspring’s health outcomes. For example, a Spanish multicenter longitudinal study reported that low (<400 μg/day) folic acid supplements use during pregnancy was associated with poorer attentional function in girls and poorer working memory in boys, and high (≥1000 μg/day) folic acid supplement was associated with a better working memory only in girls [67]. Moreover, the effects of prenatal folic acid supplementation on body weight and insulin resistance were sex-dependent in a rat experiment, which showed that maternal folic acid supplementation could increase body weight and insulin sensitivity in male offspring, but decrease body weight and increase insulin sensitivity in female offspring [56]. Similarly, our study found that maternal folic acid supplementation in pregnancy was significantly and negatively associated with obesity in preschooler girls born SGA, but not in preschooler boys born SGA.

The sex-specific effects of prenatal folic acid supplement on offspring’s health outcomes might be explained by the following reasons. First, the aforementioned rat study showed that genes related to appetite regulation, such as proopiomelanocortin, neuropeptide Y, leptin receptor, and agouti-related protein, were expressed differently in males and females [56]. Second, several researchers have suggested that different sex-specific methylation patterns might explain the sex-specific effects. For example, a trial in mice showed that a high fat diet could lead to global DNA hypomethylation, but only in females [68]. Similarly, one of our previous studies found that placenta areas mediated the positive association between DNA methylation of FGFR2 in placenta and full-term low birth weight only in girls, but not in boys [69]. Moreover, Sinclair’s sheep models showed that restricting folate in pregnancy caused 53% of changed loci of DNA methylation exclusive for males, while only 12% specific to females [70]. Third, adipose or body composition diverged between boys and girls as early as the fetus period, which might be another biological basis for the sex differences identified [71]. Further studies are needed to decipher the sex-specific responses to the maternal prenatal supplementations of nutrients and the mechanisms lying behind these sex differences.

### 4.4. Limitation

Some limitations should be considered when interpreting the results. First, our findings are based upon a retrospective observation study, so caution is required when interpreting a causal relationship. Second, all variables in this study were collected by a self-administered questionnaire, which might be affected by memory bias and so influence the validity and reliability of our measures of prenatal nutrient supplementation. Third, unfortunately we did not collect any detailed information about the dose, frequency, and period of maternal nutrients supplement, limiting our ability to fully assess the association between prenatal nutrients supplement and offspring’ obesity. Fourth, we did not collect the information on the specific vitamins that comprised the multivitamin used by the mothers during pregnancy, so we were unable to identify the associations between specific combinations of vitamin supplementation and offspring’ obesity. Fifth, we were unable to assess the level of relevant nutrients (e.g., folic acid, vitamins, iron, etc.) ingested from diet during pregnancy, and so we could not control for their influence on our findings [52]. Sixth, although BMI measurement is a standard method of categorizing obese and non-obese children, it is imprecise in measuring the degree and location of body fat [72]. As such, other measures, such as bioelectric impendence and skin fold thickness, should be included in future studies. Notably, different definitions of SGA and obesity may lead to the different classification of some participants, which would undoubtedly affect the results. The differences in definitions should be considered when comparing our results with other studies. Seventh, we have excluded 902 pairs (10.11%) for missing information, which might have led to selection bias. Eighth, obesity is a complex disorder caused by multiple factors, and we acknowledged other potential confounders, such as paternal obesity, maternal diet, and genetic factors, which were not available for this study sample, and may disturb the true association between prenatal supplement of nutrients and preschool obesity. Lastly, data collection was limited to the Longhua District of Shenzhen, and so, consequently, it is possible there may be some limits to the generalizability of our findings to other regions due to cultural differences in diet (e.g., folic acid fortification cereal in North America).

## 5. Conclusions

In summary, prenatal supplementation of folic acid was found to be associated with a decreased risk of obesity in preschool girls born SGA. This effect was modified by prenatal supplementations of multivitamin and iron. These findings support public health programs that encourage appropriate prenatal maternal folic acid supplementation to reduce obesity levels in SGA girls.

## Figures and Tables

**Table 1:** Comparison of demographic characteristics between obese and non-obese children born SGA.

Characteristics	Total	Obesity, N (%)		*t*/*χ*^2^	*p*
		Yes	No		
Total	8016	702 (8.76)	7314 (91.24)	-	-
Age [(Mean ± SD) (years)]	4.85 ± 0.84	4.89 ± 0.83	4.85 ± 0.84	−1.200	0.230
Sex				15.152	<0.001
Male	4863	474 (67.52)	4389 (60.01)		
Female	3153	228 (32.48)	2925 (39.99)		
Current weight of child [(Mean ± SD) (kg)]	18.07 ± 3.64	24.32 ± 5.12	17.47 ± 2.81	−56.287	<0.001
Current height of child [(Mean ± SD) (cm)]	108.34 ± 7.69	108.51 ± 9.27	108.33 ± 7.53	−0.614	0.539
Current BMI of child [(Mean ± SD) (kg/m^2^)]	15.33 ± 2.18	20.47 ± 2.29	14.84 ± 1.38	−96.150	<0.001
Birthweight [(Mean ± SD) (kg)]	2.72 ± 0.41	2.71 ± 0.50	2.72 ± 0.40	0.469	0.639
Gestational age at birth [(Mean ± SD) (weeks)]	39.65 ± 1.90	39.58 ± 2.38	39.65 ± 1.85	0.962	0.336
Maternal age [(Mean ± SD) (years)]	28.44 ± 4.27	28.64 ± 4.41	28.42 ± 4.25	−1.310	0.190
Paternal age [(Mean ± SD) (years)]	30.55 ± 4.83	30.79 ± 4.92	30.52 ± 4.82	−1.418	0.156
Maternal prepregnancy obesity				7.856	0.005
No	7897	683 (97.29)	7214 (98.63)		
Yes	119	19 (2.71)	100 (1.37)		
Maternal prepregnancy BMI [(Mean ± SD) (kg/m^2^)]	20.31 ± 2.81	20.87 ± 3.22	20.26 ± 2.76	−5.486	<0.001
Gestational hypertension				2.651	0.266
No	7743	683 (97.43)	7060 (96.74)		
Yes	204	12 (1.71)	192 (2.63)		
Uncertain	52	6 (0.86)	46 (0.63)		
Mode of delivery				0.373	0.542
Vaginal delivery	5848	1324 (61.07)	3539 (60.52)		
Cesarean delivery	2168	844 (38.93)	2309 (39.48)		
Maternal marital state				2.251	0.134
Married	7943	692 (98.58)	7251 (99.14)		
Others *	73	10 (1.42)	63 (0.86)		
Paternal marital state				0.567	0.451
Married	7935	693 (98.72)	7242 (99.02)		
Others *	81	9 (1.28)	72 (0.98)		
Maternal education level				3.345	0.188
Junior high school or lower	1350	135 (19.23)	1215 (16.61)		
High school	1628	143 (20.37)	1485 (20.30)		
College or higher	5038	424 (60.40)	4614 (63.08)		
Paternal education level				10.542	0.005
Junior high school or lower	1177	131 (18.66)	1046 (14.30)		
High school	1687	150 (21.37)	1537 (21.01)		
College or higher	5152	421 (59.97)	4731 (64.68)		
Household income [(Chinese Yuan)]				10.785	0.005
0–9999	1369	151 (21.51)	1218 (16.65)		
10,000–29,999	4613	386 (54.99)	4227 (57.79)		
≥30,000	2034	165 (23.50)	1869 (25.55)		
Single child				37.421	<0.001
Yes	2893	179 (25.50)	2714 (37.11)		
No	5123	523 (74.50)	4600 (62.89)		

* Including divorced, remarried, spouse loss, unmarried.

**Table 2:** The association of prenatal maternal supplementation of the three micronutrients with obesity in preschoolers born SGA.

Nutrients Supplementation	Total, N = 8016	Obesity, N (%)	AOR (95% CI) ^a^
Folic acid			
No	567	71 (12.52)	1.00
Yes	7449	631 (8.47)	0.72 (0.55, 0.93) *
Multivitamin			
No	4873	454 (9.32)	1.00
Yes	3143	248 (7.89)	0.89 (0.75, 1.05)
Iron			
No	4914	453 (9.22)	1.00
Yes	3102	249 (8.03)	0.91 (0.77, 1.07)

^a^: adjusted for child’s sex, child’s age, mode of delivery, parents’ age at the childbirth, maternal prepregnancy BMI, marital status, parents’ education level, family income, and single child or not in models. * *p <* 0.05.

**Table 3:** The combination effect of maternal supplementation of nutrients on obesity in preschoolers born SGA.

Nutrients Supplementation		AOR (95% CI) ^a^	IOR (95% CI) ^a^	RERI (95% CI) ^a^	AP (95% CI) ^a^
Folic acid	Multivitamin		1.07 (0.40, 2.84)	0.08 (−0.76, 0.92)	0.12 (−1.14, 1.39)
No	No	1.00			
No	Yes	0.86 (0.33, 2.25)			
Yes	No	0.73 (0.55, 0.97) *			
Yes	Yes	0.67 (0.50, 0.90) **			
Folic acid	Iron		0.69 (0.35, 1.39)	−0.38 (−1.28, 0.52)	−0.54 (−1.78, 0.69)
No	No	1.00			
No	Yes	1.31 (0.67, 2.58)			
Yes	No	0.77 (0.57, 1.04)			
Yes	Yes	0.70 (0.52, 0.96) *			

^a^: adjusted for child’s sex, child’s age, mode of delivery, parents’ age at the childbirth, maternal prepregnancy BMI, marital status, parents’ education level, family income, and single child or not in models. * *p* < 0.05, ** *p* < 0.01.

**Table 4:** The association of prenatal maternal supplementation of the three micronutrients with obesity in preschoolers born SGA in stratified analysis by sex.

Male				Female			
Nutrients Supplementation	Total, *N =* 4863	Obesity, *N* (%)	AOR (95% CI) ^a^	Nutrients Supplementation	Total, N = 3153	Obesity, N (%)	AOR (95% CI) ^a^
Folic acid				Folic acid			
No	361	46 (12.74)	1.00	No	206	25 (12.14)	1.00
Yes	4502	428 (9.51)	0.78 (0.56, 1.09)	Yes	2947	203 (6.89)	0.57 (0.36, 0.90) *
Multivitamin				Multivitamin			
No	2999	319 (10.64)	1.00	No	1874	135 (7.20)	1.00
Yes	1864	155 (8.32)	0.83 (0.67, 1.02)	Yes	1279	93 (7.27)	1.01 (0.76, 1.34)
Iron				Iron			
No	3010	307 (10.20)	1.00	No	1904	146 (7.67)	1.00
Yes	1853	167 (9.01)	0.94 (0.77, 1.15)	Yes	1249	82 (6.57)	0.85 (0.64, 1.13)

^a^: adjusted for child’s age, mode of delivery, parents’ age at the childbirth, maternal prepregnancy BMI, marital status, parents’ education level, family income, and single child or not in models. * *p <* 0.05.

**Table 5:** The combination effect of maternal supplementation of nutrients on obesity in preschoolers born SGA in stratified analysis by sex.

Sex	Nutrients Supplementation		AOR (95% CI) ^a^	IOR (95% CI) ^a^	RERI (95% CI) ^a^	AP (95% CI) ^a^
Male						
	Folic acid	Multivitamin		0.68 (0.22, 2.11)	−0.37 (−1.75, 1.02)	−0.52 (−2.45, 1.41)
	No	No	1.00			
	No	Yes	1.22 (0.40, 3.73)			
	Yes	No	0.85 (0.60, 1.20)			
	Yes	Yes	0.71 (0.49, 1.03)			
	Folic acid	Iron		0.56 (0.24, 1.28)	−0.73 (−2.08, 0.63)	−0.90 (−2.48, 0.69)
	No	No	1.00			
	No	Yes	1.66 (0.74, 3.71)			
	Yes	No	0.87 (0.60, 1.27)			
	Yes	Yes	0.81 (0.55, 1.20)			
Female						
	Folic acid	Multivitamin		3.55 (0.44, 28.50)	0.74 (0.08, 1.40)	1.34 (−0.07, 2.74)
	No	No	1.00			
	No	Yes	0.31 (0.04, 2.46)			
	Yes	No	0.50 (0.31, 0.82) **			
	Yes	Yes	0.55 (0.33, 0.92) *			
	Folic acid	Iron		1.16 (0.31, 4.33)	0.17 (−0.83, 1.17)	0.34 (−1.69, 2.37)
	No	No	1.00			
	No	Yes	0.76 (0.21, 2.76)			
	Yes	No	0.57 (0.35, 0.94) *			
	Yes	Yes	0.51 (0.30, 0.86) *			

^a^: adjusted for child’s age, mode of delivery, parents’ age at the childbirth, maternal prepregnancy BMI, marital status, parents’ education level, family income, and single child or not in models. * *p* < 0.05, ** *p* < 0.01.

## Data Availability

The datasets generated and/or analyzed during the current study are not publicly available due to privacy protection of the participants, but are available from the corresponding author on reasonable request.
